# Anti-Transglutaminase 6 Antibodies in Children and Young Adults with Cerebral Palsy

**DOI:** 10.1155/2014/237107

**Published:** 2014-04-02

**Authors:** Reidun Stenberg, Marios Hadjivassiliou, Pascale Aeschlimann, Nigel Hoggard, Daniel Aeschlimann

**Affiliations:** ^1^Department of Pediatrics, Centre for Rehabilitation Research and Department of Clinical Medicine, School of Health and Medical Sciences, Örebro University, 70185 Örebro, Sweden; ^2^Department of Neurology and Department of Neuroradiology, The Royal Hallamshire Hospital, Sheffield S10 2JF, UK; ^3^Matrix Biology & Tissue Repair Research Unit, College of Biomedical and Life Sciences, School of Dentistry, Cardiff University, Cardiff CF14 4XY, UK

## Abstract

*Objectives*. We have previously reported a high prevalence of gluten-related serological markers (GRSM) in children and young adults with cerebral palsy (CP). The majority had no enteropathy to suggest coeliac disease (CD). Antibodies against transglutaminase 6 (anti-TG6) represent a new marker associated with gluten-related neurological dysfunction. The aim of this study was to investigate the prevalence of anti-TG6 antibodies in this group of individuals with an early neurological injury resulting in CP. *Materials and Methods*. Sera from 96 patients with CP and 36 controls were analysed for IgA/IgG class anti-TG6 by ELISA. *Results*. Anti-TG6 antibodies were found in 12/96 (13%) of patients with CP compared to 2/36 (6%) in controls. The tetraplegic subgroup of CP had a significantly higher prevalence of anti-TG6 antibodies 6/17 (35%) compared to the other subgroups and controls. There was no correlation of anti-TG6 autoantibodies with seropositivity to food proteins including gliadin. *Conclusions*. An early brain insult and associated inflammation may predispose to future development of TG6 autoimmunity.

## 1. Introduction

Patients with cerebral palsy (CP) are characterized by abnormal muscle tone, coordination problems, and delay in motor development leading to difficulties in gait, balance, and involuntary movements. Muscle spasticity is very common. Associated conditions include epilepsy, dysarthria, cognitive impairment, and feeding problems, which may result in difficulties in weight gain and growth [1, 2] particularly for the most severely affected CP groups (tetraplegic and dyskinetic subgroups). The aetiology of the brain damage causing CP is among others mainly hypoxia due to asphyxia leading to ischemic brain injury. Another cause could be intrauterine infection [3–5].

Since coeliac disease (CD) is a common cause of poor growth, we previously analysed our cohort of CP patients for IgA and IgG-antibodies against gliadin and transglutaminase 2 (TG2) and found a high prevalence of seropositive patients. The highest prevalence was for IgG-AGA 36 and 61%, respectively [6, 7] which is of low diagnostic value for CD [8]. The majority of these patients also did not have enteropathy on routine histological or extended immunohistochemical examination of small bowel biopsies [6, 9, 10]. However, a number of patients tested positive for TG2 IgA (7/99) and/or had circulating antibodies to deamidated gliadin peptides (DGP; 7/40 tested), accepted markers in CD diagnosis [6, 10]. The seropositive CP patients had a significantly lower weight, height, and body mass index (BMI) and they were also more severely handicapped, according to gross motor function classification (GMFCS) [11].

Increasing numbers of studies have reported that gluten-related disorders include extraintestinal manifestations, for example, involving skin (dermatitis herpetiformis) and brain (gluten Ataxia) [12–15]. Such manifestations may occur in the absence of overt gastrointestinal involvement and patients may be seronegative for anti-TG2 IgA autoantibodies, the commonly used diagnostic marker for CD [16].

TG2 is the autoantigen in CD, whilst TG3 is the autoantigen in dermatitis herpetiformis, a blistering skin disease with gluten-induced granular IgA-deposits in the papillary dermis [12]. Another enzyme in the transglutaminase family that is primarily expressed in the central nervous system is transglutaminase 6 (TG6) [17]. We have demonstrated the presence of circulating anti-TG6 antibodies in adults with gluten Ataxia, independent of intestinal involvement [16]. This may suggest a bias of the immune response towards different TG isozymes in extraintestinal manifestations (i.e., TG6 in gluten Ataxia and TG3 in dermatitis herpetiformis).

The aim of this study was to investigate the prevalence of antibodies against TG6 in patients with CP.

## 2. Material and Methods

### 2.1. Participants

#### 2.1.1. CP-Group

The study recruited 99 children and young adults with CP living in the Swedish county council of Värmland and Örebro. In Sweden children and young adults with CP are treated at specialized rehabilitation centers where they can obtain specific support such as medical care, physiotherapy, speech, occupational, and psychological therapy until they have finished school. In some cases they are 24 years of age before they are referred to the adult clinic.

There were 46 girls and 53 boys. At the time of enrolment the age ranged from 18 months to 24 years (median age 11 years). There was no comorbidity for CD such as diabetes mellitus or dermatitis herpetiformis at the time of inclusion. The diagnosis of CP subgroups was based on studies by Mutch et al. [18, 19]. A functional assessment of each child was made on the basis of the Gross Motor Function Classification System (GMFCS), graded I–V, where GMFCS V represent the most severe disability [11]. Medical data of the CP-group were reviewed retrospectively in medical files (RS). Thirteen children had percutaneous gastrostomy (PEG) but also received some gluten containing food orally. Eleven children had treatment for gastroesophageal reflux disease (GERD).

#### 2.1.2. Controls

36 children, aged 2–18 years (19 boys and 17 girls), were included in the serological analysis as controls. These children had been investigated with laboratory analyses for allergy but were found negative. No further clinical information about the children was available.

### 2.2. Serology

Data on IgG and IgA AGA and TG2, DGP antibodies and HLA testing were available from our previous studies [6, 10] as well as the data of antibodies against dietary proteins [7].

None in the CP-group had IgA deficiency (<0.07 g/L).

### 2.3. TG6 Antibody ELISA

Detection of antibodies to TG6 by ELISA followed our previously published protocol with minor modifications [16]. Briefly, full-length human TG6 produced in SF9 cells was obtained from Zedira (Darmstadt, Germany). Results with this antigen were comparable to those obtained with recombinant TG6 produced in house in* E. coli* (data not shown) [16]. TG6 was diluted to 2 *μ*g/mL in 20 mM Tris/HCl, 300 mM NaCl, pH 7.6 immediately prior to use and applied to high-capacity protein binding 96-well plates (Immulon 2HB; Thermo Electron, Waltham, MA, USA) overnight at 4°C. All subsequent incubations were conducted at room temperature. Nonspecific binding was blocked by incubation with 3% BSA (immunoassay grade, Sigma 05477) in TBS (20 mM Tris/HCl, pH 7.4, 150 mM NaCl) for 1 h. Patient sera were diluted 1 : 100 in 1% BSA in TBS and any protein aggregates present removed by centrifugation at 10,000 ×g for 5 min prior to being applied to coated plates. All binding steps were carried out for 90 min and followed by five rinses with TBS/0.01% Tween 20. Antibody binding was detected by incubation with peroxidase-conjugated affinity purified anti-human IgA (Jackson ImmunoResearch, West Grove, PA, USA; 109-035-011, diluted 1 : 1000 in 1% BSA/TBS) or anti-human IgG (Dako, Carpinteria, CA, USA; P0214, diluted 1 : 250 in 1% BSA/TBS). The reaction was finally developed for 2 h using 5 mM 5-amino-2-hydroxybenzoic acid/NaOH, pH 6.0, 0.005% H_2_O_2,_ as a peroxidase substrate solution and stopped by addition of an equal volume of l M NaOH to each well. After 15 minutes, the absorbance at 490 nm was measured.

All serum samples were analysed in duplicate on wells containing antigen or only BSA, included on the same plate. The BSA only background was subtracted from values for antigen. Units were calculated from a series of standards (0, 1, 3, 10, and 100 U/mL; 1st generation assay) (Zedira) run in parallel. Results are given as the mean of two independent determinations. A measurement >14 U/mL for IgA or >34 U/mL for IgG was considered positive. Thresholds were set as the 98th percentile on a blood donor collective. As anti-BSA antibodies may be prevalent in this patient cohort which has enhanced immunity to dietary components [7] and this may potentially impact on the analysis, we have evaluated all samples also in a commercial assay without background subtraction (Zedira). There was good agreement between the results of the two ELISA methods (supplementary figure).

### 2.4. Ethics

The study was approved by Regional Ethical Review Board in Uppsala. Participants or parents, as appropriate, gave written informed consent.

### 2.5. Statistical Analyses

All data were analyzed using the Statistical Package for the Social sciences (SPSS) program, version 15. Differences between groups were evaluated with Pearson's *χ* ^2^ test in cross tabulations and when appropriate Fisher's Exact Test. An independent *t*-test was used for body mass index (BMI) (standard deviation, SD), height (SD), and weight (SD), using 2-tailed significance.

*P* < 0.05 was considered significant.

## 3. Results

### 3.1. Serological Analysis for Anti-TG6 Autoantibodies

**Figure 1:**
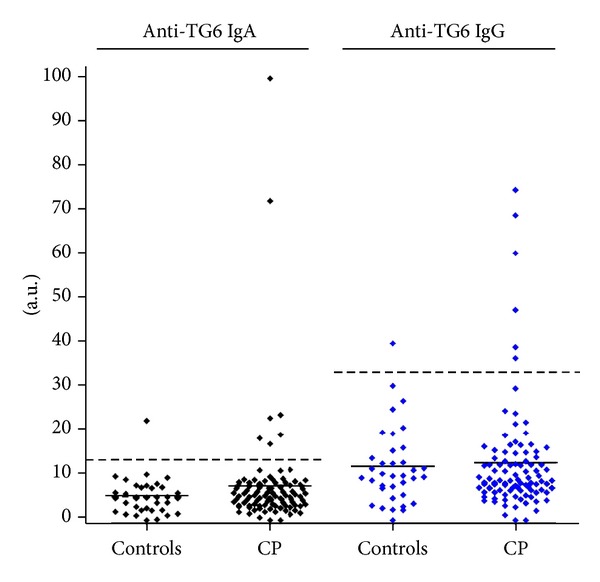
Analysis of serum for antibodies against transglutaminase type 6 (TG6) by ELISA. Relative concentration of antibodies in children (*n* = 96) with cerebral palsy (CP) and controls (*n* = 36) is given in arbitrary units. Bolded line represents the mean titre of the group and dotted line the threshold for a positive test.

**Figure 2:**
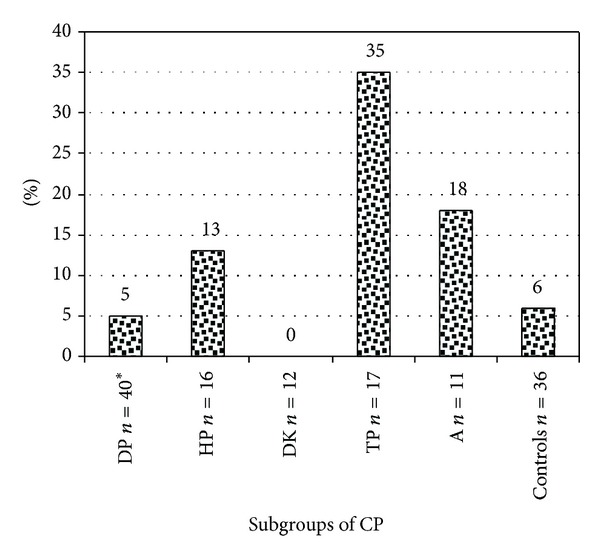
Percentage of patients testing positive for IgA/IgG antibodies to TG6 in different CP subgroups and in a control group. The tetraplegic subgroup compared to the other CP subgroups (*P* = 0.006) and to controls (*P* = 0.01); *n = 3 missing for TG6 antibody analysis. HP: CP-Hemiplegia, DP: CP-Diplegia, TP: CP-Tetraplegia, DK: CP-Dyskinesia, and A: CP-Ataxia.

Sera from 96 patients with CP and 36 controls (children from same geographical area) were available for analysis of autoantibodies against TG6. We found elevated levels of anti-TG6 antibodies (IgG and/or IgA) in 12/96 (13%) in the CP-group and 2/36 (6%) in the control group (*P* = 0.35). (Figure 1) However, a positive test for TG6 antibodies was significantly more frequent in the tetraplegic subgroup of CP, 6/17 (35%) compared to the control group 6% (*P* = 0.01). We also found statistical significance when the tetraplegic subgroup was compared to the other CP subgroups (*P* = 0.006) (Figure 2). IgA anti-TG6 antibodies were found in 7/96 (7%) compared to 1/36 (3%) in the control group. (*P* = 0.45) IgG anti-TG6 antibodies were found in 6/96 (6%) compared to 1/36 (3%) in the control group (*P* = 0.67). One child had elevated levels of both IgA and IgG to TG6.

This CP cohort has been tested for CD as reported previously. [6, 10] There was an association between anti-TG6 antibodies and IgA antibodies to TG2 (*P* = 0.04) but not for any of the other gluten-related serological markers analysed (anti-TG2 IgG or AGA IgA/IgG) (Table 1(b)). Five of the twelve patients with CP that tested positive for anti-TG6 antibodies were negative for all gluten-related serological markers. Also, there was no correlation between anti-TG6 antibody titres and the presence of other indicators of CD (Table 1(b)). Eleven of the twelve individuals with TG6 positivity had previously been tested for the coeliac HLA type (DQB1 typing) and 6/11 were positive for HLA-DQ2 and/or HLA-DQ8, and a further 2 carried one-half of the DQ2 heterodimer (DQ7, *α*5 subunit) which is known to confer susceptibility to CD [21]. A further patient carries the rare HLA-DQ9 which may also confer susceptibility according to recent evidence [22].

### 3.2. Clinical Data of Patients Testing Positive for Anti-TG6 Antibodies

The clinical data of the 12 CP patients positive for anti-TG6 antibodies are summarized in Table 1(a). The median age was significantly higher in the TG6 positive group, 14.4 years compared to 10.7 years in the TG6 negative group (*P* = 0.021).

The majority was born at term (8/12) and had asphyxia. Five had epilepsy-requiring medication. There was no significant difference in weight (*P* = 0.318) or BMI (*P* = 0.987) between TG6 antibody positive and negative patients. There was, however, a significant difference in height between the 2 groups. The children and young adults with CP positive for anti-TG6 were shorter (*P* = 0.021). As height correlates to the degree of disability this may reflect a more severe disability, consistent with a higher prevalence of anti-TG6 antibodies in the tetraplegic subgroup [1].

Seven of these patients had previously been investigated on clinical grounds using MRI or CT and the results were reviewed as part of this study (NH). Brain malformation was seen in one child and traumatic injury in another two. Developmental malformations or defects as a consequence of ischemia were seen in 3 cases. In one child with Ataxia no significant abnormalities were found (Table 1(a)). As gastrointestinal dysfunction typically seen in the most severe forms of CP may impact on gut permeability and lead to enhanced immunity to food-derived antigens, we further evaluated whether a correlation existed between TG6 autoantibodies and indicators of feeding difficulties. There was no significant difference for either the group that had treated GERD or assisted feeding (PEG) with regard to TG6 antibody positivity (Table 2). In contrast, anti-gliadin antibodies previously identified [6] showed a strong association with PEG and also correlated with patient weight and BMI (Table 2).

## 4. Discussions

The rational for the study was that anti-TG6 antibodies have been described in the context of neurological manifestations in gluten-related disorders and may identify gluten sensitivity in patients serologically negative for anti-TG2 antibodies [23].

There was a significantly higher prevalence of TG6 antibodies in the subgroup of patients with the most severe form of CP but not in the CP group as a whole. Furthermore, a positive correlation of TG6 antibodies with TG2 IgA antibodies but not to other antibodies tested is in keeping with a shared/overlapping mechanistic origin of these autoantibodies as has been suggested [24].

CP is a heterogeneous condition and is likely to have a number of different causes. The most common cause is an ischemic event in the immature brain. Bax et al. showed that magnetic resonance imaging (MRI) abnormalities were detectable in 88% of 351 children with CP [3]. The major findings seen on MRI in the tetraplegic subgroup was white matter damage of immaturity, including periventricular leukomalacia (PVL) (35.1%) and to a lesser extent cortical lesions. The finding of PVL was previously thought to be a typical damage seen in premature-born children. However, this neuroimaging study [3] has shown that white matter damage can be a common finding also among term infants and that in such cases the white matter loss tends to be more extensive. The localisation and severity of the brain lesions depend on the timing of the event in the immature brain and differ between the subgroups of cerebral palsy [3] and so also the clinical outcome of the lesion.

The weight is in general lower in the children with CP compared to normal children and some children with CP require food intake in an alternative way, that is, by a percutaneous gastrostomy (PEG), to gain weight due to their oral and gastrointestinal dysfunction. The most severe forms of CP are in the subgroups of Dyskinesia (DK) and Tetraplegia (TP). These patients also suffer most from gastrointestinal disturbances and have the lowest weight gain of the CP subgroups. In our recently published study from the same cohort of patients, we found mainly IgG-antibodies not only to gluten but also to other food proteins (lactoglobulin and casein) in the DK and TP subgroups of CP who also had the lowest weight [7]. It is likely that these patients have increased intestinal permeability allowing entrance of undigested proteins across the gut barrier into the circulation [25] and therefore have enhanced immunity to food-derived antigens. Such immune reactivity to dietary constituents has also been reported in studies of underweight children from underdeveloped countries [25, 26]. However, we could not find a significant association between lower body weights or the use of PEG and the presence of anti-TG6 autoantibodies; in contrast, AGA antibodies significantly correlated with low weight and assisted feeding (PEG).

There was a significantly higher prevalence of TG6 autoantibodies in the tetraplegic subgroup of patients compared to controls and the other subgroups. In fact there was no antibody response to TG6 in the other subgroup of CP with severe deficiencies, the dyskinetic group. The reason for that is not clear. One possibility could be that the type of brain damage in the tetraplegic subgroup (extensive white matter involvement) may predispose to future development of an immune reaction against TG6. Indeed white matter abnormalities on brain MR imaging are a common finding in patients with CD and headaches [8, 27–30]. The hypothesis of a primary brain insult leading to sensitisation to gluten is supported by a recent study demonstrating increased prevalence of CD in patients with previous head injury [31].

TGs are a family of structurally and functionally related cross-linking enzymes. In mice, TG6 expression is associated with neurogenesis in CNS development and in the mature brain, in neurons in regions associated with motor function including the cerebral cortex and cerebellum [17]. Antibodies isolated from CD patients frequently react with human and mouse neurons. Such antibodies are not the predominant anti-TG2 IgA but either directed to TG6 or cross-reactive between the closely related isozymes, TG2, TG3 and TG6 [32]. Intraventricular injection of such patient-derived TG-specific immunoglobulins in mice induced Ataxia-like deficits [32]. These data not only indicate that autoantibodies to TG6 could be a marker of brain lesions but that such antibodies may play a part in pathogenesis. The role of TG6 in cerebellar functioning has recently been further highlighted by the identification of mutations in the human gene encoding TG6, causing autosomal dominant spinocerebellar Ataxia [33, 34].

## 5. Conclusion

This study group of children with CP does not have a higher prevalence of celiac disease than expected in the regional population but they do have more frequent immunoreactivity to gluten and other dietary food components compared to matched controls [6, 7]. Here, we report significantly increased prevalence of anti-TG6 autoantibodies in the tetraplegic subgroup of patients with CP. This antibody response seems not to be correlated with low body weight and the associated immune response to food constituents. The aetiology of anti-TG6 antibodies in this CP subgroup remains unclear, but the results could support the hypothesis of a primary brain insult leading to TG6 autoimmunity.

## Supplementary Material

Sera were analysed for anti-TG6 antibodies using two different ELISA assays (Supplementary Figure). There was good agreement between these two methods in that 12/96 patients tested positive in the in house assay and 13/96 in a commercial assay with an overlap of 10 patients between the assays. We have also tested whether the results from the commercial assay would substantially alter the results with regards to the CP subgroups. The TP group remains the most prevalent in terms of TG6 antibody positivity, and the association is significant.
Supplementary Figure: Comparison of different ELISA methods for assessment of TG6 antibodies in serum. The in-house assay was performed as outlined in Materials and Methods and involved subtraction of the absorbance of a blank surface (no antigen) from that of a TG6-coated surface for calculation of relative antibody titres. The Zedira ELISA (IgA: 0312GE00; IgG 0412GE00) was carried out according to the manufacturer's instructions and is based on absorbance measurement on antigen-coated surface only. The data for each assay is the mean of two or more determinations. The dotted line indicates the threshold for a positive test.

## Figures and Tables

**Table:** (a)

Patients sex/age (—/yr)	IQ N = normal MR = mental retardation	Diagnosis/subdiagnosis of CP	GMFCS	CP etiology	Neonatal data W = gestational age when born	Brain MRI/CT/US	Epilepsy and treatment
F/17	N	AU/ A	I	Unknown	PN	MRI, normal	—
M/18	MR	TP	V	Asphyxia	PN Asphyxia at birth	US, wide ventricles, small subependymal bleeding	—
M/4	MR	TP	IV-V	Traumatic injury 3 months of age	PN	CT, focal infarct, cortical, subcortical, and basal ganglia damage	Lamotrigine
M/12	N	A	II	Hydrops fetalis	Hydrops fetalis CS; W 28	MRI, cortical and subcortical damage	—
F/18	MR	TP	V	Asphyxia	CS; W 40	CT, focal infarct	—
F/18	N	HF/HP	II	Left cerebral infarction at 1 years of age	PN	MRI and CT, cortical and subcortical damage, and hydrocephalus	—
M/20	MR	TP	V	Asphyxia	CS; W 40	NA	—
F/7	N	HP	I	Asphyxia	CS; W 32	NA	—
F/14	MR	DP	III	Asphyxia	W 26. Asphyxia at birth	NA	Carbamazepine
F/17	MR	HC/ HP	II	Brain malformation at birth	PN	MRI, brain malformation	Valproate
M/20	MR	TP	V	Asphyxia		NA	Valproate
M/9	MR	TP	V	Traumatic injury 1 years of age	PN	CT, focal infarct	Valproate, lamotrigine, and vigabatrin

**Table:** (b)

Patients sex/age (—/yr)	IgA anti-TG6 cutoff value >14 U/mL	IgG anti-TG6 cutoff value >34 U/mL	IgA AGA	IgG AGA	IgA TG2	IgG TG2	HLA DQ-type	IgA/IgG DGP cutoff >20 U/mL	Small bowel biopsies	GI symptoms
F/17	17.3	—	—	Pos.	—	—	7.8	—	Positive DR staining grade 1	NA
M/18	18.5	—	Pos.	—	Pos.	NA	6.8	—	Positive DR staining grade 2	—
M/4	19.4	—	—	—	—	—	5	NA	NA	—
M/12	23.9	—	—	Pos.	—	Pos.	6	—	NA	Oral dysfunction
F/18	23.1	—	—	Pos.	Pos.	Pos.	2.5	26	IgA deposits and *αβ*/*γδ* IEL's, DR staining grade 2	C
F/18	72.4	—	—	—	—	—	7	NA	NA	Unspecific abdominal pain
M/20	>100	39.2	—	—	—	—	2.9	NA	NA	GERD, C, D
F/7	—	36.7	—	—	—	—	6.9	NA	NA	—
F/14	—	47.6	—	Pos.	Pos.	Pos.	6.7	32	NA	GERD
F/17	—	60.5	—	—	—	—	2	—	NA	—
M/20	—	69.1	Pos.	—	—	—	NA	—	NA	GERD, PEG, C
M/9	—	74.8	—	Pos.	—	Pos.	6.8	—	NA	PEG, C

Abbreviations: A: Ataxia; HP: Hemiplegia; TP: Tetraplegia; DP: Diplegia; AU: Autism; HC: Hydrocephalus; HF: Heart Failure; GERD: gastroesophageal reflux disease; PN: partus normalis and born term; CS: Caesarean section; C: Constipation; N: normal; MRI: magnetic resonance imaging; CT: computer tomography; US: Ultrasound; PEG: percutaneous endoscopic gastrostomy; GMFCS: Gross Motor Function Classification System, graded I–V; D: Diarrhoea; NA: not applicable; Pos: positive; IEL: intraepithelial lymphocytes.

**Table 2:** Correlation between indicators of feeding problems and immunity to TG6 and to AGA analysed previously (*n* = 99)* [6].

	AGA positive 41/99* (41%)	AGA negative 58/99* (59%)	*P* value	TG6 antibody positive 12/96 (12.5%)	TG6 antibody negative 84/96 (87.5%)	*P* value
Weight	−1.960 SD	−1.00 SD	*P* = 0.016	−1.917 SD	−1.304 SD	*P* = 0.318
BMI	−1.175 SD	−0.136 SD	*P* = 0.006	−0.528 SD	−0.537 SD	*P* = 0.987
PEG *n* = 13	10/13	3/13	*P* = 0.005	2/13	11/13	*P* = 0.664
GERD *n* = 11	6/11	5/11	*P* = 0.348	3/10^♦^	7/10^♦^	*P* = 0.111

*90 children age < 18 year were published in the referred paper. Here we have calculated with the whole cohort, *n* = 99, since we have the data available.

^*♦*^1 missing for analysis of TG6-antibodies.

SD: weight ± standard deviation (SD), also referred to as weight *z*-scores, standardized to the Swedish general population by age and sex and based on 3,650 healthy children [20]. GERD: gastroesophageal reflux disease; PEG: percutan endoscopic gastrostomy; AGA: IgA and IgG gliadin antibodies TG6: transglutaminase 6.

## Abbreviations

A: CP-Ataxia
AU: Autism
CP: Cerebral palsy
CT: Computer tomography
DP: CP-Diplegia
DK: CP-Dyskinesia
GMFCS: Gross Motor Function Classification System, graded I–V
GERD: Gastroesophageal reflux
GRSM: Gluten-related serological markers (IgG/IgA-TG2, IgG/IgA-AGA)
HP: CP-Hemiplegia
MRI: Magnetic resonance imaging
PEG: Percutaneous endoscopic gastrostomy
TP: CP-Tetraplegia
US: Ultrasound.
