# Supplementary material for: Anti-Transglutaminase 6 Antibodies in Children and Young Adults with Cerebral Palsy
Source: Autoimmune Dis. 2014 Apr 2;2014:237107. doi: 10.1155/2014/237107 (PMC3996887; doi:10.1155/2014/237107)
Supplement: Supplementary file 1 — Sera were analysed for anti-TG6 antibodies using two different ELISA assays (Supplementary Figure). There was good agreement between these two methods in that 12/96 patients tested positive in the in house assay and 13/96 in a commercial assay with an overlap of 10 patients between the assays. We have also tested whether the results from the commercial assay would substantially alter the results with regards to the CP subgroups. The TP group remains the most prevalent in terms of TG6 antibody positivity, and the association is significant. Supplementary Figure: Comparison of different ELISA methods for assessment of TG6 antibodies in serum. The in-house assay was performed as outlined in Materials and Methods and involved subtraction of the absorbance of a blank surface (no antigen) from that of a TG6-coated surface for calculation of relative antibody titres. The Zedira ELISA (IgA: 0312GE00; IgG 0412GE00) was carried out according to the manufacturer's instructions and is based on absorbance measurement on antigen-coated surface only. The data for each assay is the mean of two or more determinations. The dotted line indicates the threshold for a positive test. [file 237107.f1.pdf]

Supplementary Figure: Comparison of different ELISA methods for assessment of TG6 antibodies in serum. The in-house assay was performed as outlined in Materials and Methods and involved subtraction of the absorbance of a blank surface (no antigen) from that of a TG6-coated surface for calculation of relative antibody titres. The Zedira ELISA (IgA: 0312GE00; IgG 0412GE00) was carried out according to the manufacturer's instructions and is based on absorbance measurement on antigen-coated surface only. The data for each assay is the mean of two or more determinations. The dotted line indicates the threshold for a positive test.

A

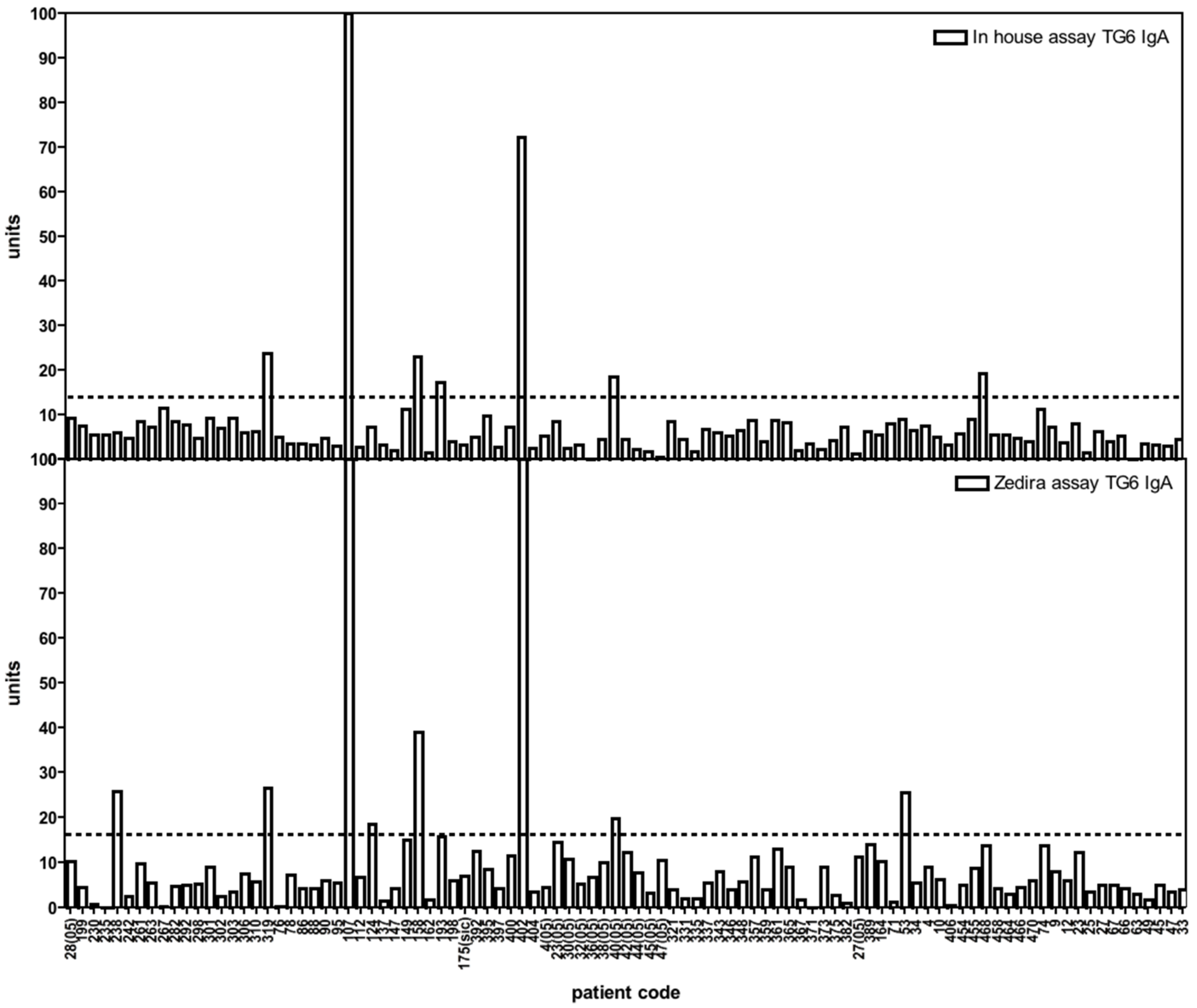

In house assay TG6 IgA
Zedira assay TG6 IgA
units
units
patient code

B

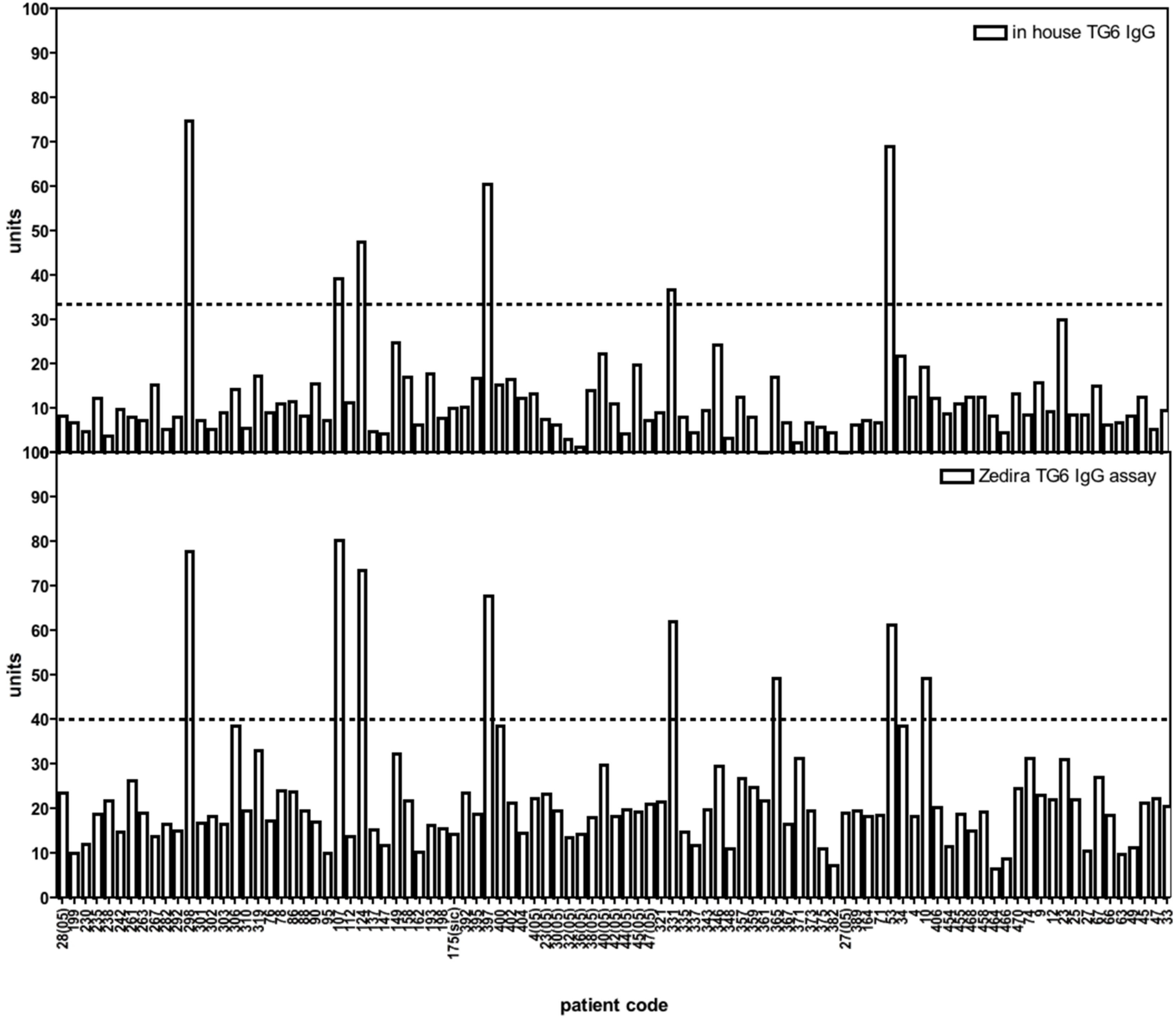
in house TG6 IgG
Zedira TG6 IgG assay
units
units
patient code
